# Supplementary figures and images for: A Phylogenetic Perspective on the Evolutionary Patterns of the Animal Interleukin-10 Signaling System
Source: Genes (Basel). 2025 Oct 22;16(11):1243. doi: 10.3390/genes16111243 (PMC12652433; doi:10.3390/genes16111243)

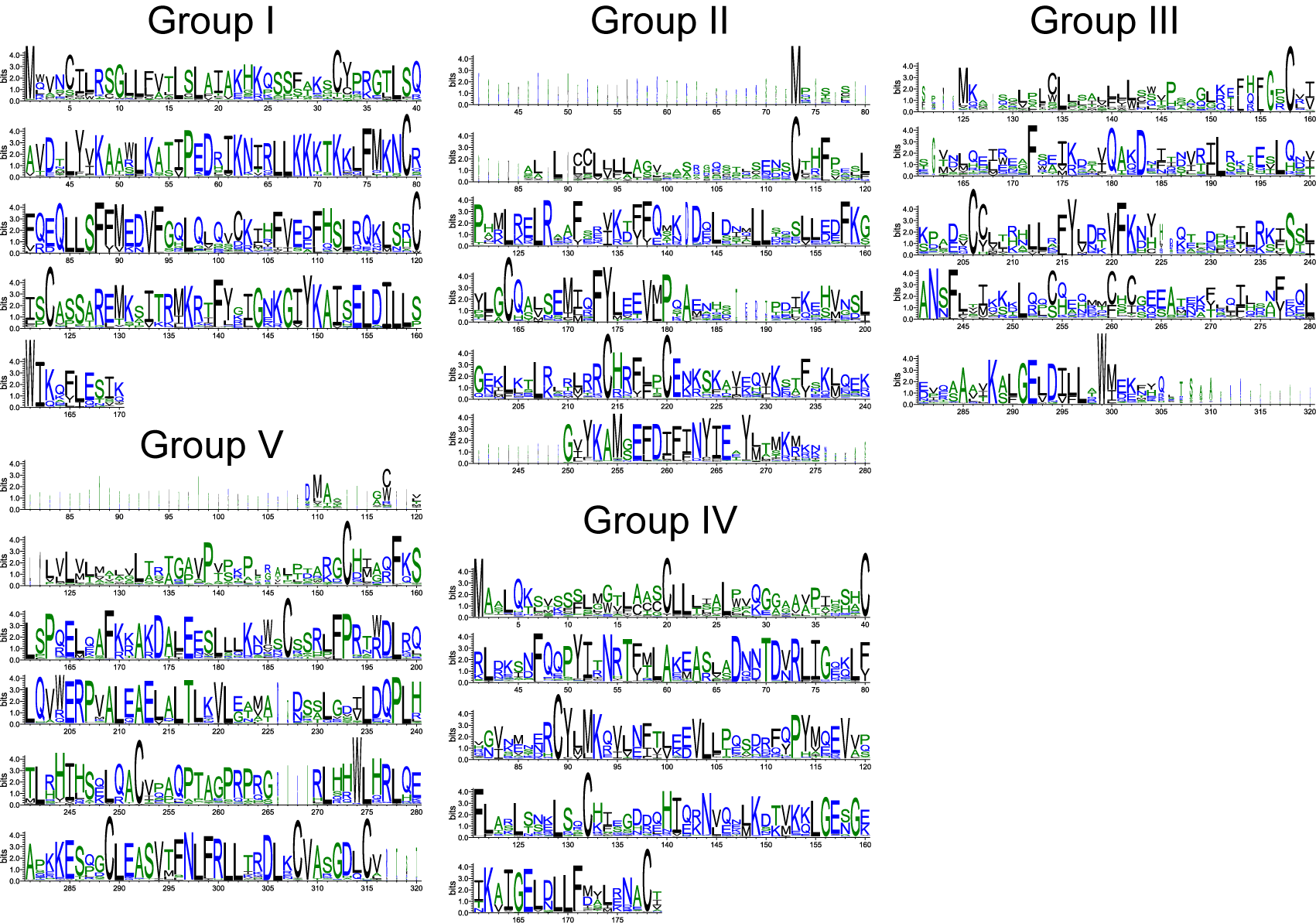

Supplement: Supplementary file 1 [file genes-16-01243-s001.zip › Supplementary Figure 1.tif]

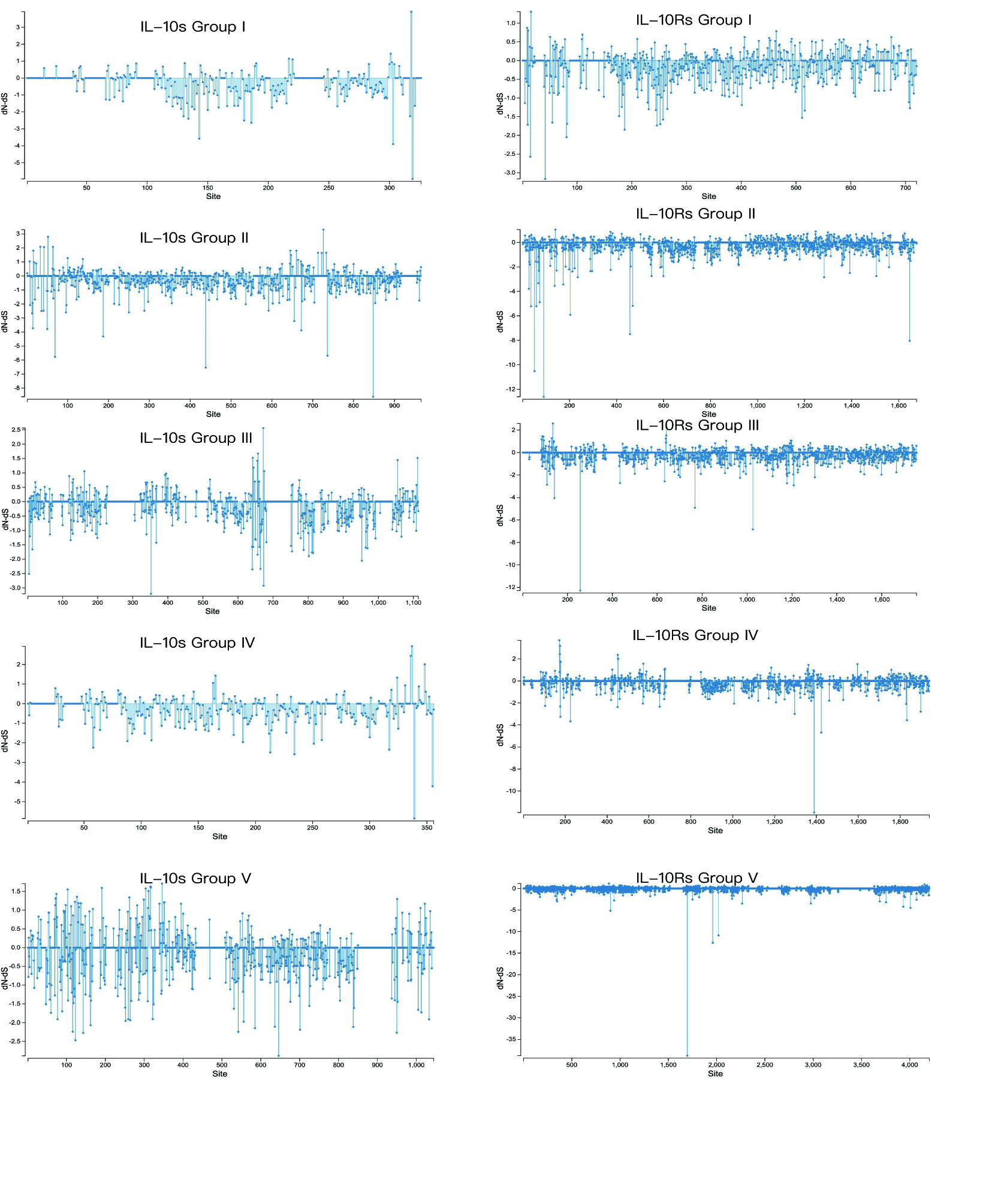

Supplement: Supplementary file 1 [file genes-16-01243-s001.zip › Supplementary Figure 2.tif]
